# Validation of Roussouly classification in predicting the occurrence of adjacent segment disease after short-level lumbar fusion surgery

**DOI:** 10.1186/s10195-023-00744-0

**Published:** 2024-01-13

**Authors:** Muyi Wang, Xin Wang, Hao Wang, Yifei Shen, Yong Qiu, Xu Sun, Dong Zhou, Yuqing Jiang

**Affiliations:** 1 Department of Orthopedics, Affiliated Changzhou Second People’s Hospital of Nanjing Medical University, Gehu Middle Road 68, Changzhou, 213000 Jiangsu Province China; 2 Division of Spine Surgery, Department of Orthopedic Surgery, Affiliated Drum Tower Hospital, Medical School of Nanjing University, Nanjing, Jiangsu Province China; 3 Department of Orthopedics, Changzhou No.6 People’s Hospital, Changzhou, Jiangsu Province China; 4 Changzhou Medical Center, Nanjing Medical University, Changzhou, Jiangsu Province China

**Keywords:** Adjacent segment disease, Roussouly classification, Spinopelvic sagittal alignment, Lumbar fusion, Distal lordosis

## Abstract

**Background:**

Recent studies demonstrated that restoring sagittal alignment to the original Roussouly type can remarkably reduce complication rates after adult spinal deformity surgery. However, there is still no data proving the benefit of maintaining ideal Roussouly shape in the lumbar degenerative diseases and its association with the development of adjacent segment disease (ASD). Thus, this study was performed to validate the usefulness of Roussouly classification to predict the occurrence of ASD after lumbar fusion surgery.

**Materials and Methods:**

This study retrospectively reviewed 234 consecutive patients with lumbar degenerative diseases who underwent 1- or 2-level fusion surgery. Demographic and radiographic data were compared between ASD and non-ASD groups. The patients were classified by both “theoretical” [based on pelvic incidence (PI)] and “current” (based on sacral slope) Roussouly types. The patients were defined as “matched” if their “current” shapes matched the “theoretical” types and otherwise as “mismatched”. The logistic regression analysis was performed to identify the factors associated with ASD. Finally, clinical data and spinopelvic parameters of “theoretical” and “current” types were compared.

**Results:**

With a mean follow-up duration of 70.6 months, evidence of ASD was found in the 68 cases. Postoperatively, ASD group had more “current” shapes classified as type 1 or 2 and fewer as type 3 than the non-ASD group (*p* < 0.001), but the distribution of “theoretical” types was similar between groups. Moreover, 80.9% (55/68) of patients with ASD were mismatched, while 48.2% (80/166) of patients without ASD were mismatched (*p* < 0.001). A multivariate analysis identified age [odds ratio (OR) = 1.058)], 2-level fusion (OR = 2.9830), postoperative distal lordosis (DL, OR = 0.949) and mismatched Roussouly type (OR = 4.629) as independent risk factors of ASD. Among the four "theoretical" types, type 2 had the lowest lumbar lordosis, DL, and segmental lordosis. When considering the "current" types, current type 2 was associated with higher rates of 2-level fusion, worse DL, and greater pelvic tilt compared with other current types.

**Conclusions:**

DL loss and mismatched Roussouly type were significant risk factors of ASD. To decrease the incidence of ASD, an appropriate value of DL should be achieved to restore sagittal alignment back to the ideal Roussouly type.

*Level of Evidence*: Level 4.

## Introduction

Spinal fusion surgery has been a standard of care for lumbar degenerative diseases refractory to conservative treatment and can produce satisfactory clinical results [1]. However, lumbar arthrodesis may increase biomechanical stress on the levels neighboring fused segments, which could possibly cause early adjacent segment disease (ASD) [2]. Symptomatic ASD frequently results in deterioration of the clinical outcome and requirement of further surgical treatment.

With the goal of establishing potential preventive methods, numerous studies are carried out to investigate the risk factors for ASD. Recently, increasing attention has been paid to the role of spinopelvic sagittal malalignment in the development of ASD. Maintaining or restoring “normal sagittal alignment” is of paramount importance in the lumbar fusion surgery [3]. Although few studies have demonstrated that pelvic incidence (PI) minus lumbar lordosis (LL, PI − LL) < 10° is a useful predictor for ASD, this simple formula have limitations [4]. It is controversial where the idea range of PI − LL should lie, since thresholds varies with different populations [4, 5]. Arbitrary use of an absolute numeric value for the evaluation of sagittal alignment may be misleading [6]. Emerging evidences have demonstrated that radiographic targets of surgery should be tailored to individual [4, 7].

Previously, Roussouly et al. [8] defined four types of spinal shapes in healthy population based on sacral slope (SS) and the shape of lordosis. Then, they described the possible evolution of these “normal” types under degenerative conditions [9]. Subsequent studies demonstrated that restoring sagittal alignment to the original type can remarkably reduce complication rates after adult spinal deformity surgery [10]. Additionally, a few studies have evaluated the influence of different Roussouly sagittal profiles on the outcome of patients who received lumbar decompression or fusion surgery [11, 12]. However, there is still no data proving the benefit of maintaining ideal Roussouly shape in the lumbar degenerative diseases and its association with the development of ASD. Thus, this study was performed to validate the usefulness of Roussouly classification to predict the occurrence of ASD after short-level lumbar fusion surgery.

## Materials and methods

### Patients

After the approval of Institutional Review Board, a retrospective review of one database comprising patients with lumbar degenerative diseases between January 2009 and January 2018 was performed. The patient enrollment criteria were as follows: (1) age between 40 and 80 years at the time of the index surgery, (2) treated with L4–5 or L3–5 fusion and screw fixation using the conventional posterior approach, and (3) had a follow-up duration of more than 5 years with a complete set of outcome measures and radiological examinations. The exclusion criteria were as follows: (1) had ASD observed at the caudal segment or at both cranial and caudal segments; (2) had a prior history of spinal surgery, trauma, tumor or infection; (3) the Cobb angle of lumbar curve exceeding 10° on the coronal plane; (4) diagnosed as acute or delayed deep surgical site infection after primary surgery; and (5) had a type 3 + anteverted pelvis (AP) sagittal shape.

Every patient was treated with laminectomy decompression, pedicle screw instrumentation, and fusion. Transforaminal lumbar interbody fusion (TLIF) procedures were generally performed at each level [1]. In a few patients with 2-level fusion, TLIF procedures were performed at one level. For another level with a less degenerated disc and no evidence of foraminal or central canal stenosis, posterolateral intertransverse process fusion was carried out instead of TLIF [13]. Standing posteroanterior and lateral radiographs were taken preoperatively and at each follow-up visit. The computed tomography (CT) scans and the magnetic resonance imaging (MRI) were performed before surgery. In addition, the MRI and flexion (F)–extension (E) lateral radiographs were obtained at the latest follow-up.

### Radiographic evaluation

Preoperative disc degeneration of cranial adjacent segment on MRI and facet joint degeneration of cranial adjacent segment on CT were evaluated according to the previous proposed criteria [14, 15]. The intervertebral disk height of cranial adjacent segment was measured on neutral lateral radiographs [16]. The following spinopelvic parameters were collected before surgery and at 3-month follow-up: (1) PI; (2) SS; (3) pelvic tilt (PT) (4) LL: the angle subtended by the superior end plate line of L1 and S1; (5) distal lordosis (DL): the angle between the upper endplate of L4 and S1; (6) sagittal vertical axis (SVA): the perpendicular distance between the C7 plumb line and posterior–superior endplate of the S1; (7) lordosis distribution index (LDI): the percentage of DL contribution to the LL; and (8) segmental lordosis (SL): the lordosis between the upper instrumented vertebra and the lower instrumented vertebra.

Based on the previous work of Pizones et al. [17, 18], patients were classified by both “theoretical” and “current” Roussouly types. The “theoretical” classification relied on PI to divide patients into four types: type 1 and 2 corresponded to PI < 45º, type 3 to PI between 45º and 60º, and type 4 to PI > 60º [19]. This classification provided the ideal sagittal profile for each patient: the idea SS, lumbar apex, inflexion point, and number of vertebrae in lordosis (NVL) [18]. Then, the “current” types were evaluated using the previous proposed criteria: type 1 and 2 corresponded to SS < 35º, type 3 to SS between 35º and 45º, and type 4 to SS > 45º [19]. The lumbar apex, inflexion point, NVL, and sagittal shape were also recorded. These parameters were especially important to differentiate type 1 and type 2 shapes, as PI and SS values were shared by them [9, 19]. According to the above parameters, the patients were classified as “matched” if their postoperative “current” shape matched the “theoretical” type and otherwise as “mismatched”.

In the current study, all radiographic parameters were measured twice at an interval of 1 week by a well-trained observer, and the mean of both measurements was used for subsequent analysis. The values of intraobserver reproducibility were calculated and quantified by the intraclass correlation coefficient (ICC) for all measurements. There were strong intraobserver agreements for all parameters, as all ICCs exceeded 0.8.

### ASD definition

The diagnosis of radiological degeneration was made when radiographs and MRI showed one or more of the following pathologies at a cranial segment firstly adjacent to fusion that were not present preoperatively: (1) narrowing of disc height of > 10% or development of slippage > 3 mm on a upright lateral radiograph [3, 20, 21], (2) a sagittal translation of more than 3 mm or intervertebral angle change of more than 10° on F–E modality [22, 23], or (3) advancement in disc degeneration, disc herniation or spinal canal stenosis evaluated by MRI [21, 24]. ASD was defined as newly developed or aggravated radiological degeneration adjacent to the fused levels caused recurrent clinical symptoms, such as low back and leg pain, numbness, or intermittent claudication during the follow-up period [21, 23, 25].

### Statistical analyses

Statistical analyses were performed using SPSS version 25.0 (IBM Corp., Armonk, NY). The unpaired *t*-test was used to determine the differences in the continuous data between ASD and non-ASD groups. A chi-square test or Fisher’s exact test, depending on the number of subjects involved, was used for categorical data analysis. A *p* value of less than 0.05 was considered statistically significant. Variables with *p* < 0.1 in the univariate analysis were included in the multivariate analysis with a forward stepwise method to evaluate adjusted associations between potential variables and ASD development.

The relationships between postoperative spinopelvic parameters and age, as well as PI, were analyzed using the Pearson or Spearman correlation analysis, and simple linear regressions were simultaneously conducted. In a subanalysis, patients were stratified by both “theoretical” and “current” Roussouly types. A one-way analysis of variance (ANOVA) test was used to evaluate differences in the spinopelvic parameters among types.

## Results

### Patients

A total of 234 consecutive patients were enrolled in this study. The average age at the index surgery was 60.1 years (range, 41–78 years). The fusion level was L4–5 in 118 and L3–5 in 116 cases, respectively. With a mean follow-up duration of 70.6 months (range, 60–121 months), evidence of ASD was found in the 68 cases. The pathologies of radiological degeneration included progression of retrolisthesis in 28 patients, spinal stenosis in 24 patients, and aggravation of disc herniation in 16 patients. To date, 31 patients had received revision surgery due to the ASD, while the rest were relieved by conservative treatment.

As shown in the Table 1, the characteristics of the ASD and non-ASD groups did not differ statistically in terms of sex, Pfirrmann grade, facet grade, disc height, body mass index (BMI), and follow-up duration, but the age at the index surgery in the ASD group was significantly higher than that in the non-ASD group (*p* < 0.001). Meanwhile, the differences in the fusion level and etiology between groups were statistically significant (all *p* < 0.05). Regrading medical comorbidities, the difference was only detected in the osteoporosis (*p* = 0.043).

**Table 1:** Comparison of demographic and clinical characteristics between the non-ASD and ASD groups

	Non-ASD	ASD	*P* value
Number of patients	166	68	–
Age at surgery, years	58.5 ± 10.5	64.0 ± 7.6	< 0.001
Sex			
Male	66 (39.8%)	36 (52.9%)	0.065
Female	100 (60.2%)	32 (47.1%)	
Fusion level			
L4–5	102 (61.4%)	16 (23.5%)	< 0.001
L3–5	64 (38.6%)	52 (76.5%)	
Etiology			
Isthmic spondylolisthesis	26 (15.7%)	2 (2.9%)	0.001
Degenerative spondylolisthesis	56 (33.7%)	12 (17.6%)	
Disc herniation	22 (13.3%)	10 (14.7%)	
Spinal stenosis	16 (9.6%)	8 (11.8%)	
Multiple	46 (27.7%)	36 (52.9%)	
Pfirrmann grade (cranial)			
1	–	–	0.768
2	20 (12.0%)	6 (8.8%)	
3	88 (53.0%)	38 (55.9%)	
4	58 (34.9%)	24 (35.3%)	
5	–	–	
Facet grade (cranial)			
0	24 (14.5%)	16 (23.5%)	0.189
1	80 (48.2%)	24 (35.3%)	
2	40 (24.1%)	16 (23.5%)	
3	22 (13.3%)	12 (17.6%)	
Disc height (cranial), mm	9.2 ± 1.8	9.6 ± 2.0	0.136
BMI, kg/m^2^	26.3 ± 3.5	26.9 ± 4.5	0.330
Follow-up, months	68.9 ± 12.8	70.7 ± 15.6	0.402
Medical comorbidities			
Coronary artery disease	24 (14.5%)	10 (14.7%)	0.961
Diabetes mellitus	42 (25.3%)	20 (29.4%)	0.518
Hypertension	108 (65.1%)	46 (67.6%)	0.705
Cerebral infarction	2 (1.2%)	2 (2.9%)	0.375
Osteoporosis	6 (3.6%)	7 (10.3%)	0.043

Values are presented as number (%) or mean ± standard deviation unless otherwise indicated

*ASD* adjacent segment disease, *BMI* body mass index

### Comparison of spinopelvic alignment between groups

There were significant differences in the preoperative LL and SVA between the ASD and non-ASD groups (all *p* < 0.05). Postoperatively, PI, SS, LL, and DL in the ASD group were lower than those in the non-ASD group (all *p* < 0.05; Table 2). The distribution of “theoretical” types was similar between the ASD and non-ASD groups, but there were more “current” shapes classified as type 1 or 2 and fewer as type 3 in the ASD group when compared with non-ASD group (*p* < 0.001). Moreover, 80.9% (55/68) of the patients who suffered ASD after surgery were mismatched, while 48.2% (80/166) of the patients without ASD had mismatched type (*p* < 0.001).

**Table 2:** Comparison of spinopelvic parameters between the non-ASD and ASD groups

	Non-ASD	ASD	*P* value
Preoperative			
PT, °	18.3 ± 7.5	17.9 ± 8.6	0.731
PI, °	51.2 ± 9.3	49.2 ± 9.9	0.128
SS, °	32.9 ± 6.9	31.3 ± 8.9	0.173
LL, °	45.1 ± 10.6	39.2 ± 15.0	0.004
DL, °	28.6 ± 8.6	26.1 ± 11.0	0.090
SL, °	19.6 ± 7.8	18.9 ± 11.1	0.663
LDI, %	65.5 ± 21.2	67.5 ± 23.0	0.519
SVA, mm	11.6 ± 36.8	28.5 ± 47.7	0.010
PI − LL			
≤ 10°	110 (66.3%)	46 (67.6%)	0.839
> 10°	56 (33.7%)	22 (32.4%)	
Postoperative			
PT, °	17.1 ± 6.0	16.4 ± 7.6	0.501
PI, °	51.7 ± 9.2	48.9 ± 9.7	0.042
SS, °	34.6 ± 6.7	32.6 ± 8.2	0.047
LL, °	48.9 ± 9.6	43.9 ± 11.1	0.001
DL, °	31.3 ± 8.2	27.4 ± 8.5	0.001
SL, °	20.8 ± 7.3	21.0 ± 6.7	0.803
LDI, %	64.9 ± 15.3	63.2 ± 16.6	0.442
SVA, mm	7.8 ± 28.7	7.8 ± 28.1	0.994
PI − LL			
≤ 10°	132 (79.5%)	48 (70.6%)	0.141
> 10°	34 (20.5%)	20 (29.4%)	
Roussouly type match			
Matched	86 (51.8%)	13 (19.1%)	< 0.001
Mismatched	80 (48.2%)	55 (80.9%)	
Theoretical sagittal profile			
Roussouly type 1	17 (10.2%)	8 (11.8%)	0.091
Roussouly type 2	15 (9.0%)	14 (20.6%)	
Roussouly type 3	102 (61.4%)	34 (50.0%)	
Roussouly type 4	32 (19.3%)	12 (17.6%)	
Current sagittal profile			
Roussouly type 1	22 (13.3%)	12 (17.6%)	< 0.001
Roussouly type 2	38 (22.9%)	36 (52.9%)	
Roussouly type 3	92 (55.4%)	12 (17.6%)	
Roussouly type 4	14 (8.4%)	8 (11.8%)	

Values are presented as number (%) or mean ± standard deviation unless otherwise indicated

*ASD* adjacent segment disease, *PT* pelvic tilt, *PI* pelvic incidence, *SS* sacral slope, *LL* lumbar lordosis, *DL* distal lordosis, *SL* segmental lordosis, *LDI* lordosis distribution index, *SVA* sagittal vertical axis

**Fig. 1:**
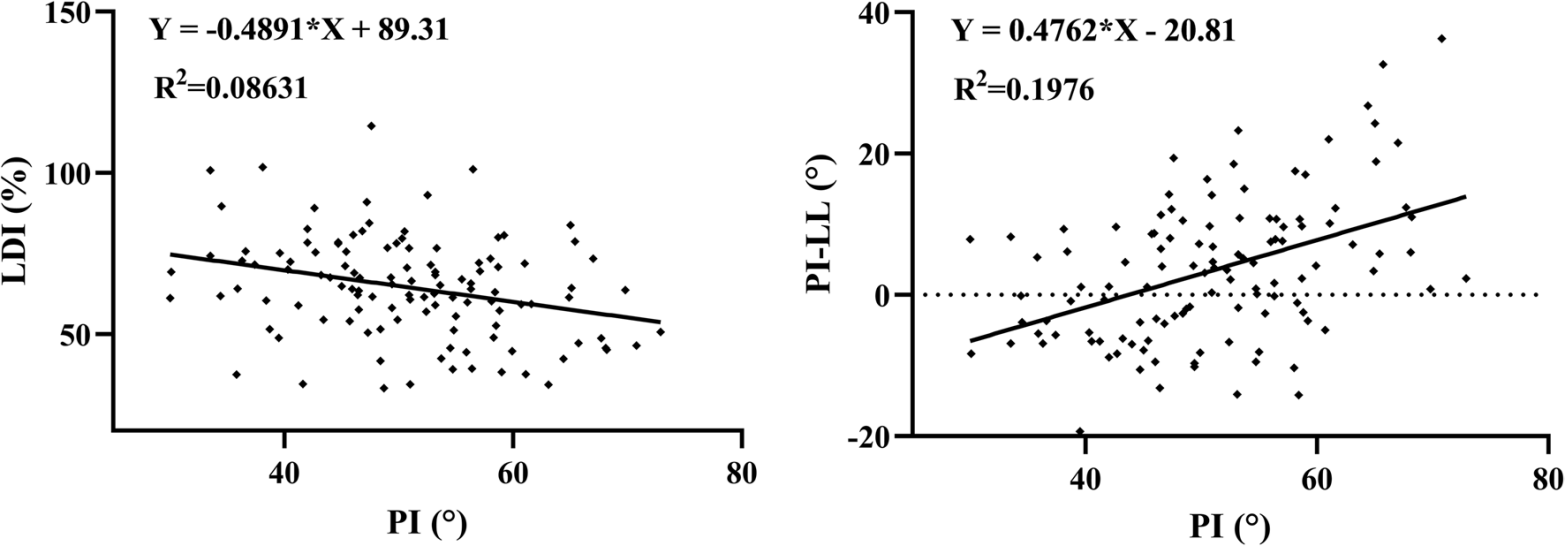
Linear regression between lumbar sagittal parameters (LDI and PI − LL) and PI. *LDI* lordosis distribution index, *PI* pelvic incidence, *LL* lumbar lordosis

Pearson or Spearman correlation tests showed that age was only correlated to SVA (*r* = 0.192; *p* = 0.003). PI was correlated to PT (*r*_*s*_ = 0.612; *p* < 0.001), SS (*r* = 0.727; *p* < 0.001), LL (*r* = 0.479; *p* < 0.001), SL (*r*_*s*_ = 0.395; *p* < 0.001), LDI (*r*_*s*_ = −0.300; *p* < 0.001), PI − LL (*r*_*s*_ = 0.418; *p* < 0.001), and SVA (*r* = 0.160; *p* = 0.014) but not DL (*r*_*s*_ = 0.098; *p* = 0.133). Linear regression analysis (Fig. 1) found a linear correlation between PI and lumbar sagittal parameters (LDI = −0.4891*PI + 89.31, R^2^ = 0.086, *p* < 0.001; PI − LL = 0.4762*PI-20.81, R^2^ = 0.198, *p* < 0.001).

### Risk factors of ASD

**Fig. 2:**
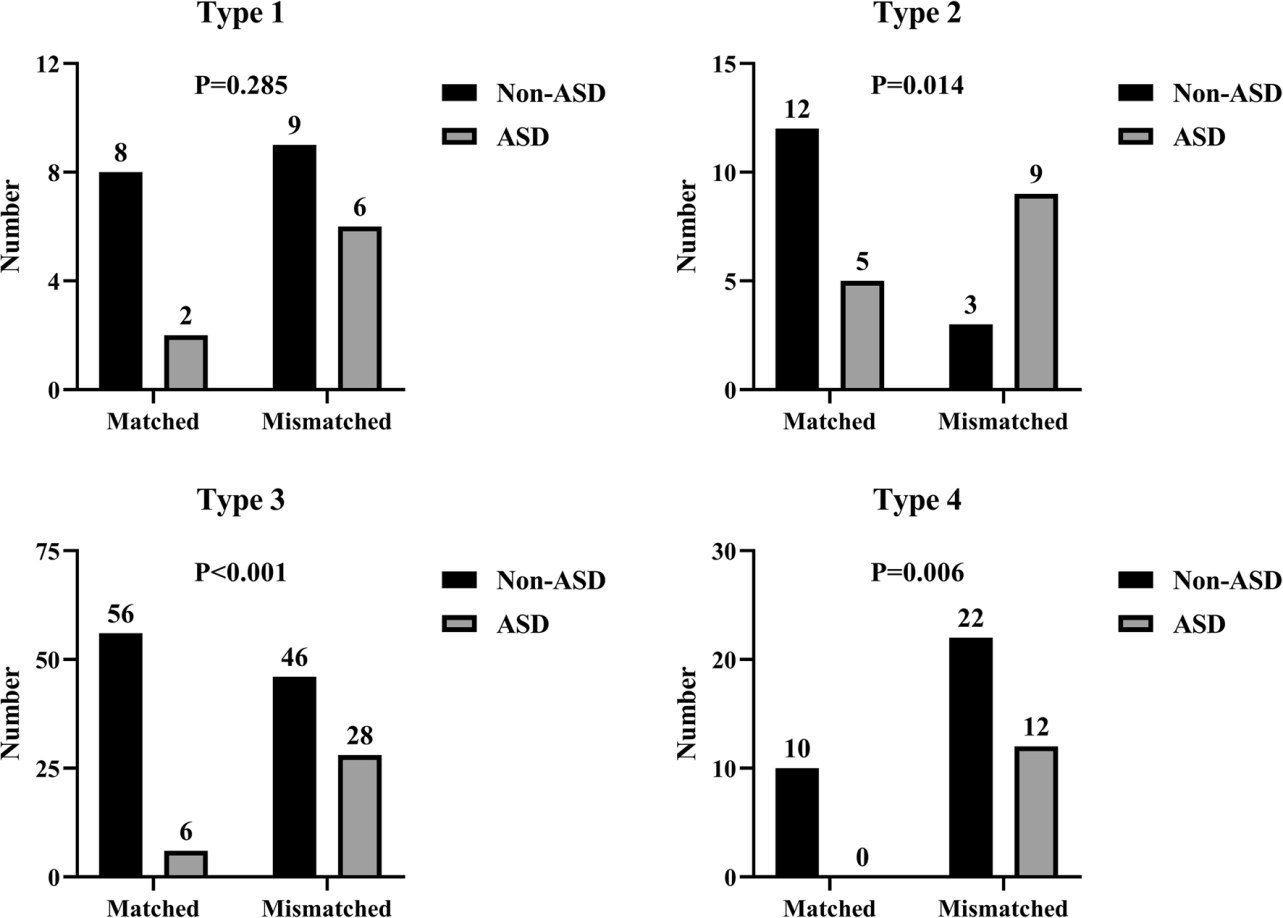
Comparison of incidence of ASD between matched and mismatched groups in the four theoretical profiles. *ASD*, adjacent segment disease

Age; sex; fusion level; etiology; osteoporosis; and postoperative PI, SS, LL, DL, and Roussouly type match were included in the multivariate analysis. The model finally chose four independent risk factors: age (OR = 1.058, 95% CI 1.013–1.105; *p* = 0.012), 2-level fusion (OR = 2.983, 95% CI 1.349–6.597; *p* = 0.007), postoperative DL (OR = 0.949, 95% CI 0.911–0.989; *p* = 0.014), and postoperative mismatched Roussouly type (OR = 4.629, 95% CI 2.239–9.570; *p* < 0.001). When patients were stratified by “theoretical” types, those who had a mismatched type were more predisposed to the occurrence of ASD than those who were matched to their ideal shape in all four types, and statistical differences were found in the type 2, 3, and 4 (Fig. 2).

### Subanalysis by Roussouly type

When considering the "theoretical" type, the differences in age and fusion level among the groups were not statistically significant. However, there were significant differences among the four theoretical types in terms of all spinopelvic parameters, except for SVA. Type 2 exhibited significantly lower values for LL, DL, and SL compared with types 1, 3, and 4 (Table 3). When considering the "current" types, the percentage of 2-level fusion in type 1 and 2 was significantly higher compared with type 3 and 4 (*p* < 0.001). Furthermore, type 2 exhibited the highest PT and the lowest values for LL, DL, and SL among the four groups. The LDI of type 2, 3, and 4 became similar and significantly lower than that of type 1. (*p* < 0.001; Table 4).

**Table 3:** Comparison of clinical data and postoperative spinopelvic parameters of four theoretical sagittal profiles

	Type 1	Type 2	Type 3	Type 4	*P* value
Age at surgery, years	62.3 ± 9.7	59.3 ± 9.0	60.7 ± 10.3	57.4 ± 9.6	0.162
Fusion level					
L4–5	10 (41.6%)	12 (40.0%)	68 (50.0%)	28 (63.6%)	0.163
L3–5	14 (58.3%)	18 (60.0%)	68 (50.0%)	16 (36.4%)	
PT, °	12.6 ± 4.2	12.3 ± 5.7	16.5 ± 5.1	23.3 ± 6.9	< 0.001
PI, °	38.9 ± 4.1	38.3 ± 4.4	51.4 ± 4.4	64.5 ± 4.0	< 0.001
SS, °	26.3 ± 4.7	26.0 ± 6.1	34.9 ± 5.0	41.2 ± 5.7	< 0.001
LL, °	42.2 ± 5.8	38.4 ± 9.9	48.6 ± 8.8	53.1 ± 11.8	< 0.001
DL, °	30.6 ± 4.6	24.8 ± 8.3	31.4 ± 7.8	30.1 ± 10.7	0.002
SL, °	19.5 ± 5.4	15.4 ± 9.0	20.8 ± 6.0	26.2 ± 7.1	< 0.001
LDI, %	73.0 ± 9.7	65.8 ± 19.3	65.2 ± 15.0	56.3 ± 14.6	< 0.001
SVA, mm	1.3 ± 28.4	5.9 ± 31.4	7.3 ± 28.4	14.2 ± 26.4	0.305
PI − LL					
≤ 10°	25 (100%)	27 (93.1%)	108 (79.4%)	20 (45.5%)	< 0.001
> 10°	0 (0%)	2 (6.9%)	28 (20.6%)	24 (54.5%)	

Values are presented as number (%) or mean ± standard deviation unless otherwise indicated

*PT* pelvic tilt, *PI* pelvic incidence, *SS* sacral slope, *LL* lumbar lordosis, *DL* distal lordosis, *SL* segmental lordosis, *LDI* lordosis distribution index, *SVA* sagittal vertical axis

**Table 4:** Comparison of clinical data and postoperative spinopelvic parameters of four current sagittal profiles

	Type 1	Type 2	Type 3	Type 4	*P* value
Age at surgery, years	61.3 ± 11.3	60.2 ± 10.8	59.4 ± 9.5	61.3 ± 8.0	0.714
Fusion level					
L4–5	12(35.3%)	24(32.4%)	68(65.4%)	14(63.6%)	< 0.001
L3–5	22(64.7%)	50(67.6%)	36(34.6%)	8(36.4%)	
PT, °	14.8 ± 5.2	18.5 ± 7.1	16.2 ± 6.1	17.4 ± 6.9	0.024
PI, °	42.3 ± 6.9	46.9 ± 9.0	53.9 ± 6.6	63.6 ± 5.3	< 0.001
SS, °	27.4 ± 4.8	28.4 ± 4.9	37.7 ± 3.0	46.2 ± 3.2	< 0.001
LL, °	41.9 ± 6.9	39.8 ± 7.5	51.7 ± 7.6	61.8 ± 7.6	< 0.001
DL, °	31.7 ± 5.4	24.1 ± 7.3	32.5 ± 7.3	36.2 ± 9.2	< 0.001
SL, °	19.7 ± 5.1	18.2 ± 7.9	21.8 ± 5.7	27.8 ± 8.0	< 0.001
LDI, %	77.0 ± 15.3	61.4 ± 17.0	63.3 ± 13.1	60.3 ± 14.1	< 0.001
SVA, mm	1.7 ± 24.5	11.2 ± 30.0	8.4 ± 28.6	2.6 ± 28.5	0.336
PI − LL					
≤ 10°	28(82.4%)	52(70.3%)	80(76.9%)	20(90.9%)	0.184
> 10°	6(17.6%)	22(29.7%)	24(23.1%)	2(9.1%)	

*PT* pelvic tilt, *PI* pelvic incidence, *SS* sacral slope, *LL* lumbar lordosis, *DL* distal lordosis, *SL* segmental lordosis, *LDI* lordosis distribution index, *SVA* sagittal vertical axis

Values are presented as number (%) or mean ± standard deviation unless otherwise indicated

## Discussion

Although the importance of spinopelvic alignment and its correlation with ASD have been validated in many studies, the “normal” alignment remains poorly defined. Previous studies have investigated the relationship between PI − LL mismatch and the occurrence of ASD. In a biomechanical study with musculoskeletal modeling, Senteler et al. [5] concluded that PI − LL ≥ 15° was a predictor of revision surgery for ASD. Rothenfluh et al. [26] showed that after receiving lumbar posterolateral fusion, patients with PI − LL ≥ 10° had a tenfold greater risk of developing ASD than controls. However, a 10-year follow-up study by Toivonen et al. [6] demonstrated postoperative PI − LL > 9° did not result in a significantly increased risk of revision for ASD. Our study also did not find a statistically significant effect of PI − LL on the rate of ASD. The patients with low PI were likely to be PI − LL match, while patients with high PI tended to be classified as mismatch. Hence, reaching the simplistic target of PI − LL match does not always prevent the occurrence of ASD. Subsequent studies proposed that sagittal realignment should take the entirety of age-related dynamic generative changes into account and determined new age-specific values for sagittal parameters, such as age-adjusted PI − LL [7]. In the current study, age was also recognized as an independent risk factor of ASD. However, our results showed that age only correlated with and SVA. Thus, it is still controversial whether age-specific sagittal parameters could be used in the assessment of ASD.

**Fig. 3:**
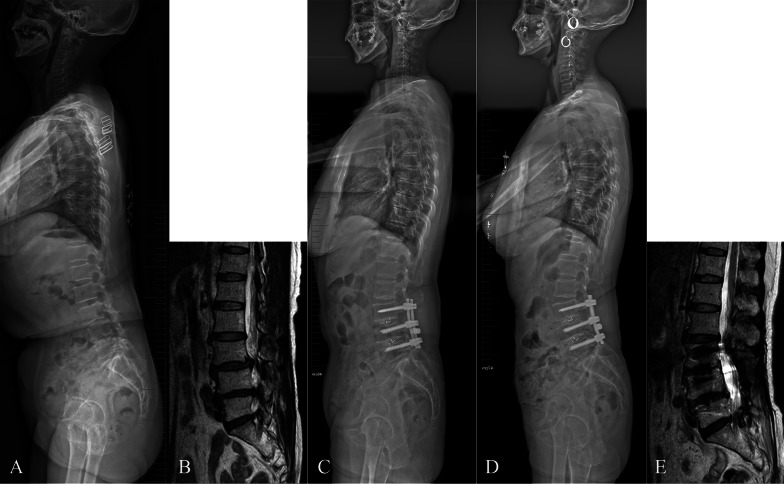
A case with mismatched Roussouly type. A 59-year-old female with lumbar degenerative spondylolisthesis and spinal stenosis at L3–4 and L4–5, the theoretical Roussouly shape was type 3 based on the PI of 55° (**A**, **B**). She underwent pedicle screw fixation from L3 to L5, and TLIF at L3–4 and L4–5. The upright lateral radiograph showed that PT was 22°, SS was 33°, LL was 37°, DL was 16°, LDI was 43%, lumbar apex was L3, inflexion point was T10 (indicating the retroverted type 2), and the sagittal profile did not match the ideal Roussouly type (**C**). At 5-year follow-up, she complained of recurrent low back pain and leg pain and numbness. The upright lateral radiograph showed hypertension and retrolisthesis at the adjacent segment, and MRI detected occurrence of L2–3 spinal stenosis (**D**, **E**). *PI* pelvic incidence, *PT* pelvic tilt. *SS* sacral slope, *LL* lumbar lordosis, *DL* distal lordosis, *LDI* lordosis distribution index, *TLIF* transforaminal lumbar interbody fusion, *MRI* magnetic resonance imaging

**Fig. 4:**
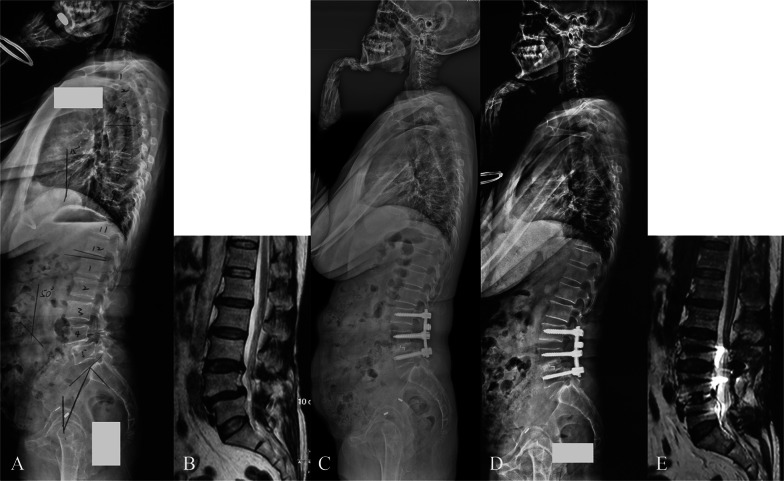
A case with matched Roussouly type. A 60-year-old female with lumbar spinal stenosis and isthmic spondylolisthesis at L3–4 and L4–5, the theoretical Roussouly shape was type 3 based on the PI of 57° (**A**, **B**). She underwent pedicle screw fixation from L3 to L5, and posterolateral inter-transverse process fusion at L3–4 and TLIF at L4–5. The upright lateral radiograph showed that PT was 20°, SS was 37°, LL was 49°, DL was 35°, LDI was 71%, lumbar apex was L4, and inflexion point was L1 (indicating the type 3 shape and a matched status, **C**). At 5-year follow-up, she reported being well. The upright lateral radiograph and MRI showed no signs of ASD (**D**, **E**). *PI* pelvic incidence, *PT* pelvic tilt, *SS* sacral slope, *LL* lumbar lordosis, *DL* distal lordosis, *LDI* lordosis distribution index, *TLIF* transforaminal lumbar interbody fusion, *MRI* magnetic resonance imaging, *ASD* adjacent segment disease

With regards to sagittal alignment, postoperative DL and mismatched Roussouly type were risk factors of ASD. Degenerative diseases frequently involve lower lumbar spine and lead to the loss of DL and anterior displacement of the axis of gravity [27]. Then, pelvic retroversion and upper lumbar hyperlordosis are recruited to keep sagittal balance [28]. Our result showed that compared with theoretical types, there was an increasing incidence of type 1 and 2 shapes in the current types, because high PI types (type 3 and 4) could evolute into retroverted types through pelvis retroversion [10]. Hyperextension of adjacent segments is another common local compensatory mechanism to limit the consequences of lumbar kyphosis on the shift of axis gravity [29]. Cranial adjacent segments are more extended to place the upper lumbar spine posteriorly for avoiding forward trunk. Due to pelvis retroversion and altered lordosis distribution, the lumbar sagittal shape and the location of lumbar apex may change, finally resulting in the degenerative evolution of original Roussouly type. If DL cannot be restored after fusion surgery, PT remains impaired and proximal lumbar levels continue to signify more extension for maintaining sagittal balance. This compensatory mechanism generates increase of stresses on posterior structures, exposes adjacent segment to the risk of retrolisthesis, and may result in accelerated degeneration [29]. Therefore, if the spinopelvic morphology is not paralleled with a corresponding ideal type, the patients will be predisposed to a greater risk for ASD (Figs. 3 and 4).

Recently, the role of DL in spinal biomechanics was noted and LDI was used to evaluate the risk of ASD development. Bari et al. [30] reported that in the patients received lumbar fusion surgery, hypolordotic lordosis maldistribution was associated with increased risk of revision surgery. Zheng et al. [27] also found patients with low LDI were at greater risk for developing ASD than those with high LDI after L4–S1 fusion for degenerative disease. However, it is not appropriate to define the range of 50–80% as optimal cutoff points of LDI, because there is a linear and negative correlation between PI and LDI. As shown in the previous studies, proximal levels were recruited to increase total lordosis as the PI values increased, but L4–S1 lordosis was nearly constant (approximately 35°) and independent of the PI [30, 31]. Our results also showed that PI was not correlated with DL, indicating that different PI values may share the same target of DL reconstruction. Additionally, due to a lower PI value in the ASD group compared with the non-ASD group, the presence of worse LDI suggested that the ASD group did not receive the optimal restoration of DL.

When stratified by theoretical types, the incidence of ASD was highest in the type 2. Subanalysis showed that both DL and LDI of all four theoretical types were worse than their ideal values. In addition, theoretical type 2 had the lowest DL and its value of LDI was comparable with that of theoretical type 3. This result may help explain why type 2 had the highest incidence of ASD. When it comes to current types, patients with theoretical type 3 or 4 who underwent 2-level fusion were more likely to evolve into current type 1 or 2, suggesting hypolordotic fusion was more common in the 2-level fusion. Similarly, DL of current type 2 was lowest among groups. The PT and LDI of current type 2 became even worse than those of current type 3, as high PI types did not receive optimal reconstruction of DL and converted into retroverted types [9]. Duan et al. [12] also reported that preoperative PT in current type 2 was higher than that of current type 3, and a decrease of PT was observed in type 2 after surgery. They concluded that pelvic retroversion was the main type of compensation in the current type 1 and 2. However, we should be aware that current patients with type 2 were composed of patients with both low PI and high PI. The capacity of pelvis retroversion is limited in the patients with low PI and hyperextension of adjacent segments may be the main compensatory mechanism [29, 32]. Different from SS, PI is a fixed value for any given individual and will not be modified by degenerative changes or spinal arthrodesis [33]. According to PI value, we can better speculate that which ideal sagittal profile the patient belongs to and set surgical goals [9, 19].

### Limitations

This study had several limitations. First, there was a lack of consideration of other possible factors that are associated with ASD. Due to incomplete data, some factors like paraspinal muscle atrophy and bone mineral density were not included. Second, the strength of our results was limited by a not-big-enough series. Concerning low ratio of some types, such as theoretical type 1 and 2, it was difficult to generalize with the limited patients. Additionally, type 3 AP was not involved, as only six patients who met inclusion criteria were identified as this type. More data are needed to draw the powerful conclusion. Finally, ASD is a time-dependent phenomenon. There remains a possibility that part of the non-ASD can evolve into ASD over time. Thus, a long-term follow-up study should be conducted to reduce the bias.

## Conclusion

In summary, loss of DL and mismatched Roussouly type were significant risk factors affecting the occurrence of ASD after short-level fusion surgery for lumbar degenerative diseases. In pathologic patients, PI is a reliable index for classifying sagittal types, rather than SS. To decrease the incidence of ASD, it is important to achieve an appropriate value and distribution of DL that restores sagittal alignment back to the ideal Roussouly type.

## Data Availability

The datasets generated and analysed during the current study are not publicly available due to the sensitivity of the data and concerns regarding privacy protection.

## Abbreviations

ASD: Adjacent segment disease
BMI: Body mass index
PI: Pelvic incidence
PT: Pelvic tilt
SS: Sacral slope
LL: Lumbar lordosis
DL: Distal lordosis
SL: Segmental lordosis
LDI: Lordosis distribution index
SVA: Sagittal vertical axis
NVL: Number of vertebrae in lordosis
TLIF: Transforaminal lumbar interbody fusion
CT: Computed tomography
MRI: Magnetic resonance imaging
OR: Odds ratio
CI: Confidence interval
ICC: Intraclass correlation coefficient
ANOVA: One-way analysis of variance
